# Genomic and Geographic Context for the Evolution of High-Risk Carbapenem-Resistant *Enterobacter cloacae* Complex Clones ST171 and ST78

**DOI:** 10.1128/mBio.00542-18

**Published:** 2018-05-29

**Authors:** Angela Gomez-Simmonds, Medini K. Annavajhala, Zheng Wang, Nenad Macesic, Yue Hu, Marla J. Giddins, Aidan O’Malley, Nora C. Toussaint, Susan Whittier, Victor J. Torres, Anne-Catrin Uhlemann

**Affiliations:** a Department of Medicine, Division of Infectious Diseases, Columbia University Medical Center, New York, New York, USA; b Department of Medicine Microbiome & Pathogen Genomics Core, Columbia University Medical Center, New York City, New York, USA; c Department of Microbiology, New York University, New York, New York, USA; d New York Genome Center, New York, New York, USA; e Department of Pathology and Cell Biology, Clinical Microbiology Laboratory, Columbia University Medical Center, New York, New York, USA; Louis Stokes Veterans Affairs Medical Center

**Keywords:** antimicrobial resistance, bacterial evolution, bacterial genomics, carbapenem resistance, *Enterobacter cloacae*

## Abstract

Recent reports have established the escalating threat of carbapenem-resistant Enterobacter cloacae complex (CREC). Here, we demonstrate that CREC has evolved as a highly antibiotic-resistant rather than highly virulent nosocomial pathogen. Applying genomics and Bayesian phylogenetic analyses to a 7-year collection of CREC isolates from a northern Manhattan hospital system and to a large set of publicly available, geographically diverse genomes, we demonstrate clonal spread of a single clone, ST171. We estimate that two major clades of epidemic ST171 diverged prior to 1962, subsequently spreading in parallel from the Northeastern to the Mid-Atlantic and Midwestern United States and demonstrating links to international sites. Acquisition of carbapenem and fluoroquinolone resistance determinants by both clades preceded widespread use of these drugs in the mid-1980s, suggesting that antibiotic pressure contributed substantially to its spread. Despite a unique mobile repertoire, ST171 isolates showed decreased virulence *in vitro*. While a second clone, ST78, substantially contributed to the emergence of CREC, it encompasses diverse carbapenemase-harboring plasmids, including a potentially hypertransmissible IncN plasmid, also present in other sequence types. Rather than heightened virulence, CREC demonstrates lineage-specific, multifactorial adaptations to nosocomial environments coupled with a unique potential to acquire and disseminate carbapenem resistance genes. These findings indicate a need for robust surveillance efforts that are attentive to the potential for local and international spread of high-risk CREC clones.

## IMPORTANCE

Carbapenem-resistant Enterobacter cloacae complex (CREC) has emerged as a formidable nosocomial pathogen. While sporadic acquisition of plasmid-encoded carbapenemases has been implicated as a major driver of CREC, ST171 and ST78 clones demonstrate epidemic potential. However, a lack of reliable genomic references and rigorous statistical analyses has left many gaps in knowledge regarding the phylogenetic context and evolutionary pathways of successful CREC. Our reconstruction of recent ST171 and ST78 evolution represents a significant addition to current understanding of CREC and the directionality of its spread from the Eastern United States to the northern Midwestern United States with links to international collections. Our results indicate that the remarkable ability of E. cloacae to acquire and disseminate cross-class antibiotic resistance rather than virulence determinants, coupled with its ability to adapt under conditions of antibiotic pressure, likely led to the wide dissemination of CREC.

## INTRODUCTION

Carbapenem-resistant *Enterobacteriaceae* (CRE) represent a substantial threat to modern health care, challenging our present antibiotic armamentarium and increasing mortality and health care costs, particularly among chronically ill and immunocompromised patients (1). In the United States, Klebsiella pneumoniae bacteria, particularly the widespread health care-associated ST258 clone, have accounted for the majority of CRE infections since their initial detection in the early 2000s (2). However, reports of recent studies from throughout the United States have documented the expanding distribution of carbapenem-resistant Enterobacter cloacae complex (CREC) (3–11), while the incidence of carbapenem-resistant K. pneumoniae resistance has remained stable or has declined in some areas (7, 10, 12). Overall, the genomic background of CREC is characterized by high clonal diversity (7, 8). However, accumulating evidence suggests the widespread distribution and epidemic potential of two high-risk CREC clones, ST171 and ST78 (4–7). In most studies, ST171 and ST78 isolates harbored the plasmid-encoded K. pneumoniae carbapenemase (KPC), while diverse resistance mechanisms were detected in other clonal backgrounds (7, 8). Taken together, these data suggest that the emergence of CREC has a complex history, driven in part by its ability to acquire and maintain *bla*_KPC_-harboring plasmids.

Whole-genome sequencing (WGS) has demonstrated the development of CREC clonal outbreaks superimposed on a background of diverse multidrug-resistant (MDR) lineages. A WGS study of MDR E. cloacae in the United Kingdom and Ireland revealed substantial heterogeneity among patterns of clonal spread and emergence of antibiotic resistance but included very few carbapenem-resistant isolates (13). WGS analysis of a localized CREC outbreak in Minnesota and North Dakota was consistent with a clonal outbreak of ST171 associated with an IncFIA plasmid harboring *bla*_KPC-3_ (6). More recently, Chavda et al. explored the phylogenetic structure of geographically diverse carbapenem-resistant and carbapenem-susceptible *Enterobacter* spp., demonstrating diverse mechanisms of resistance driven largely by horizontal transfer of *bla*_KPC_-harboring plasmids followed by clonal spread (8). However, to date, little has been known about the timeline of the recent evolution of CREC and its dominant clones in the context of the introduction of carbapenems in the mid-1980s. Additional gaps in knowledge, including the unique genetic features of ST171 and ST78 and their potential impact on the success of these two high-risk clones, could have important clinical and infection control implications. Here we aimed to elucidate the genomic epidemiology of CREC at a New York City hospital and to investigate the evolutionary relationships between ST171 and ST78 genomes in New York City and geographically diverse locations. Using whole-genome phylogenetic and phylogeographic analyses, we assessed the spatiotemporal spread and distribution of mobile genetic elements (MGEs) within ST171 and ST78 clonal sublineages. We also considered the potential contribution of resistance and virulence determinants to the recent emergence of CREC in the United States and worldwide.

## RESULTS

### Population structure of Enterobacter cloacae complex isolates at a New York City hospital.

**FIG 1:**
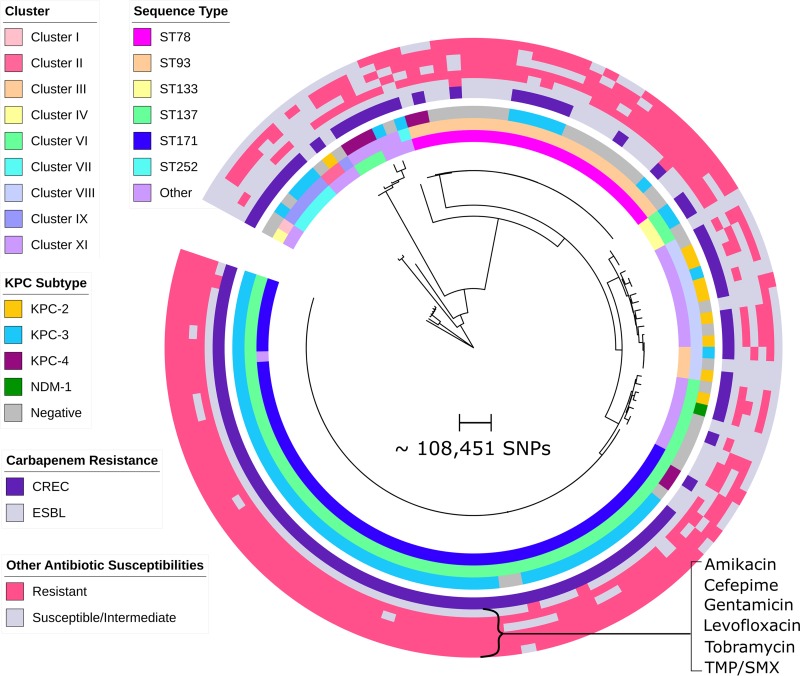
Phylogenetic tree of multidrug-resistant Enterobacter cloacae complex and clustering by MLST and *hsp60* sequencing. A maximum likelihood tree of 125 Enterobacter cloacae complex isolates was constructed based on core genome concatenated SNPs. Corresponding isolate MLST, *hsp60* gene cluster, and carbapenemase allele data are shown in the three inner circles, with groups denoted by different colors as indicated. While ST171/*hsp60* cluster VI and ST78/*hsp60* cluster III dominated among CREC and ESBL isolates, respectively, a diverse subset of isolates was also seen. Isolate resistance profiles, including carbapenem resistance phenotype (CREC versus ESBL) and resistance to 6 other major antibiotics, are shown in concentric circles on the outer periphery of the tree. ST171 isolates displayed remarkable cross-class antibiotic resistance and were uniformly susceptible only to amikacin. In contrast, ST78 CREC isolates were susceptible to levofloxacin. TMP/SMX, trimethoprim-sulfamethoxazole.

To assess the diversity of 125 E. cloacae complex isolates collected at a northern Manhattan hospital system, we constructed a phylogenetic tree based on core genome single nucleotide polymorphisms (SNPs) (Fig. 1). Isolates were distributed across 30 different sequence types (STs), predominately ST171 (*n* = 60) and ST78 (*n* = 25). Most isolates belonged to *hsp60* clusters previously shown to be pathogenic in humans, including cluster VI (ST171), cluster III (ST78), and cluster VIII (14, 15). The remaining isolates were highly diverse and belonged to six different *hsp60* clusters, including several rarely associated with human infection. Within each cluster, E. cloacae complex isolates demonstrated variable phenotypic resistance to different antibiotic classes (Fig. 1), although ST171 and, to a lesser extent, ST78 demonstrated remarkable cross-class antibiotic resistance.

### Emergence and regional spread of CREC ST171.

**FIG 2:**
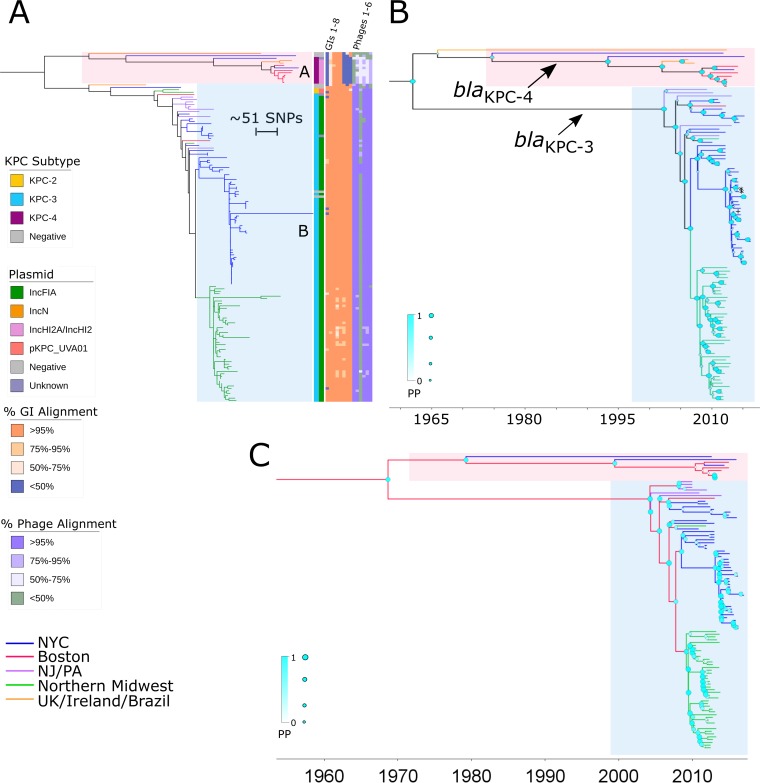
Evolution of predominant CREC clone ST171. (A) A maximum likelihood tree of ST171 core genomes constructed using RAxML, including sequences of isolates collected at our northern Manhattan hospital as well as published genomes from other geographic locations inside and outside the United States, indicates the presence of the 2 major clades (clades A and B). Geographic location is denoted by branch colors. Annotations from the inner to the outer rows include *bla*_KPC_ allele, replicon type of the putative *bla*_KPC_-harboring plasmid, and heat maps depicting percent alignment of Illumina reads to mobile genetic elements detected on ST171 reference genome 34978, including (from left to right) genomic island 1 (GI1) to GI8 and phage regions 1 to 6, for each ST171 isolate. Additional details regarding GIs and phage regions can be found in Table S4. While clade A comprised mostly *bla*_KPC-4_-harboring isolates, clade B was largely defined by *bla*_KPC-3_ carriage and included instances of local regional spread. Links to international isolates are indicated in both branches. (B and C) We also used BEAST analysis to map the phylogeny of ST171 (B), demonstrating multiple introductions of *bla*_KPC_ in this clone, as well as to further delineate the geotemporal emergence of the major ST171 clades (C). Branches are scaled by time (year-month). Support at each node is reflected by the size and color of the shapes at each junction. In panel B, arrows indicate branches with all daughter nodes containing bla_KPC-4_ or bla_KPC-3_, with the exception of 3 isolates marked with symbols (*, KPC-negative isolates; +, bla_KPC-2_-containing isolate).

In an analysis of the evolutionary history of the ST171 clone, we mapped 144 ST171 sequences to the E. hormaechei 34978 reference genome (GenBank accession number CP012165) (8) and included 84 publically available genomes to provide temporal and geographic context (see Table S2 in the supplemental material). A total of 1,659 core genome SNPs were identified and used to construct a maximum likelihood phylogenetic tree (Fig. 2A). ST171 core genomes were separated by 0 to 403 SNPs, indicating that many isolates were closely related. Using Bayesian phylogenetic analysis of isolates demonstrating evidence of clocklike evolution (*n* = 106), we estimated a substitution rate of ~2.7 SNPs per year (95% highest posterior density [HPD], 2.5 to 3.0), corresponding to a rate of 6.0 × 10^−7^ substitutions per nucleotide site per year. This estimate is comparable to that corresponding to a recently described collection of MDR E. cloacae isolates from the United Kingdom and Ireland, for which an average of 1.5 × 10^−7^ substitutions per nucleotide site per year across the genome (0.5 to 3.0 SNPs per year) was reported (13). ST171 isolates clustered into two major clades (clades A and B) which we estimate diverged prior to 1962 on the basis of the derived substitution rate (Fig. 2B).

Clade A, comprising approximately 10% of ST171 genomes, consisted of isolates originating from diverse geographic sites, including New York City, Boston, Brazil, and the United Kingdom (Fig. 2A). Isolates belonging to clade A either possessed *bla*_KPC-4_ (*n* = 9) or were *bla*_KPC_ negative (*n* = 2). *bla*_KPC-4_-harboring isolates formed a distinct branch with a common ancestor dating to 1993 (posterior probability [PP] = 0.999) (Fig. 2B), which predates the first detection of CRE in the United States, in 1996 (16). Clade B consisted of two large clusters of closely related isolates from northern Manhattan and the northern Midwest, including Minnesota and North Dakota, as well as isolates from the United Kingdom, Boston, New York City, New Jersey, Pennsylvania, and Michigan. While most ST171 CREC isolates from clade B harbored *bla*_KPC-3_ (*n* = 124), sporadic isolates possessed *bla*_KPC-2_ (*n* = 2) or were KPC negative (*n* = 4). According to results of analysis of the ancestral nodes, *bla*_KPC-3_-harboring isolates shared one recent common ancestor, which emerged prior to 2002 (PP = 1). Reconstruction of whole-genome phylogeny by the use of Gubbins software revealed that ST171 clade B was additionally defined by several small recombination events (see Fig. S1 in the supplemental material). A 70-kb recombination region encoded a total of 80 proteins, including several proteins involved in DNA replication and 7 different conjugal transfer proteins (Table S3). Within each clade, we also found evidence for the development of fluoroquinolone resistance accompanied by the acquisition and establishment of mutations in the *gyrA* (g.248C>T, g.260A>C) and *parC* (g.239G>T) genes (17).

10.1128/mBio.00542-18.1 FIG S1  Predicted recombination regions in Enterobacter cloacae complex ST171. Recombination regions in E. cloacae complex ST171 were predicted by the use of Gubbins software based on full-genome alignment of 144 ST171 isolates aligned against reference genome 34978 (GenBank accession number CP012165). Regions likely attributable to recombination which are conserved across isolates are shown in red, while recombination-related regions unique to a single isolate are shown in blue. The horizontal placement of each recombination block corresponds to the location of the region along the E. cloacae ST171 34978 reference genome. Download FIG S1, PDF file, 0.01 MB. Copyright © 2018 Gomez-Simmonds et al. 2018 Gomez-Simmonds et al. This content is distributed under the terms of the Creative Commons Attribution 4.0 International license.

Phylogeographic Bayesian analysis of U.S. isolates with defined collection locations and evidence for clocklike evolution (*n* = 107) suggests that both clade A and clade B originated in Boston and spread to other regions within the United States (PP = 0.89) (Fig. 2C). From Boston, clade A likely spread to New York City whereas clade B spread to the New York-New Jersey area (PP = 0.95) and subsequently to New York City (PP = 0.88) and the northern Midwest (PP = 0.93) between 2007 and 2008 (Fig. 2B). This was followed by local proliferation within the northern Manhattan and northern Midwestern sites with occasional cross-regional links.

### ST78 is defined by distinct CREC and extended-spectrum-beta-lactamase (ESBL)-producing sublineages.

**FIG 3:**
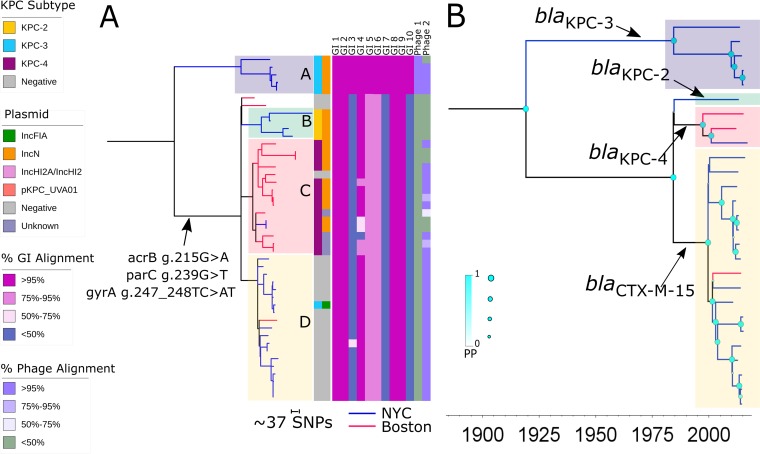
Phylogeny of ST78, including ESBL and *bla*_KPC_-harboring isolates. (A) A RAxML tree of ST78 genomes also demonstrated the presence of two major sublineages, one composed exclusively of isolates harboring *bla*_KPC-3_ (clade A) and one comprising separate ESBL and *bla*_KPC_-harboring subclades B, C, and D. Geographic location is again denoted by branch colors. Annotations from the inner to the outer rows include *bla*_KPC_ allele, replicon type of the inferred *bla*_KPC_-harboring plasmid, and percent alignment of isolate short reads to mobile genetic elements, including (from left to right) genomic island (GI) GI1 to GI10 and phage regions 1 and 2 detected on the NR0276 reference genome. Additional details regarding mobile genetic elements can be found in Table S4. (B) BEAST analysis demonstrated the initial divergence of a distinct carbapenem-resistant clade followed by separation of the predominantly ESBL and other CREC sublineages defined by carriage of different beta-lactamase genes. These two main sublineages were also distinguished by the presence of missense mutations in *gyrA* (g.247_248TC>AT), *parC* (g.239G>A), and *acrB* (g.215G>A) in clade A conferring fluoroquinolone resistance, which were not seen other ST78 isolates. Support at each node is denoted by the size and color of shapes at each junction.

Due to the lack of publicly available ST78 reference genomes, we generated a *de novo* hybrid assembly for NR0276 using Illumina short reads scaffolded with nanopore reads. The resulting NR0276 genome consisted of 2 chromosomal contigs with an average coverage of 83×, in addition to three complete plasmids assembled into single contigs (Whole Genome Shotgun Project accession number PNXT00000000). We mapped sequencing reads from 25 northern Manhattan isolates and 20 published ST78 genomes from Boston to this new reference sequence in order to reconstruct the evolutionary history of ST78 (Table S2). We identified and used 1,235 SNPs in the core chromosome to build a maximum likelihood phylogenetic tree (Fig. 3A). Pairwise SNP distances between ST78 isolates ranged from 0 to 421. We observed two major clades as confirmed through Bayesian phylogenetic analysis of isolates with clocklike evolution (*n* = 27) (Fig. 3B). On the basis of an estimated substitution rate of ~4.3 SNPs per year (95% HPD, 3.7 to 5.0), these distinct clades diverged well before 1984 (PP = 1), although a lack of earlier isolates precludes improved resolution of the date of divergence (Fig. 3B). Notably, the branch lengths separating these two clades and the length of time since divergence could not be linked to a major recombination event (Fig. S2).

10.1128/mBio.00542-18.2 FIG S2  Predicted recombination regions in Enterobacter cloacae complex ST78. Recombination regions in E. cloacae complex ST78 were predicted by Gubbins based on full-genome alignment of 45 locally collected and publicly available ST78 isolates aligned against the NR0276 *de novo* hybrid-assembly reference genome (GenBank accession no. PNXT00000000). Regions likely attributable to recombination which are conserved across isolates are shown in red, while recombination-related regions unique to a single isolate are shown in blue. The horizontal placement of each recombination block corresponds to the location of the region along the E. cloacae ST78 NR0276 reference genome. Download FIG S2, PDF file, 0.01 MB. Copyright © 2018 Gomez-Simmonds et al. 2018 Gomez-Simmonds et al. This content is distributed under the terms of the Creative Commons Attribution 4.0 International license.

Clade A, consisting of approximately 10% of the ST78 isolates (*n* = 5), was composed exclusively of CREC isolates from northern Manhattan harboring *bla*_KPC-3_ (Fig. 3A). The second, larger clade, which included isolates from both New York and Boston, consisted of several distinct subclades (subclades B, C, and D, Fig. 3A) defined by beta-lactamase carriage. Subclade B was composed of New York isolates containing *bla*_KPC-2_ (*n* = 4), while subclade C (*n* = 15) was defined by *bla*_KPC-4_ carriage and included both New York and Boston isolates. Subclade D, consisting of carbapenem-susceptible isolates (*n* = 16) harboring the ESBL *bla*_CTX-M-15_ gene, was interspersed with three sporadic carbapenem-resistant isolates, of which two were carbapenemase negative and one demonstrated uptake of *bla*_KPC-3_. Unlike the isolates in clade A, isolates in subclades B, C, and D were resistant to fluoroquinolones and were found to have missense mutations in the *gyrA* (g.247_248TC>AT), *parC* (g.239G>T), and *acrB* (g.215G>A) genes (17).

*bla*_KPC-3_-harboring ST78 CREC clade A had a most recent common ancestor dated at 1985 as suggested through Bayesian reconstruction (PP = 0.999) (Fig. 3B). Low sample size precluded confidence in the dating of the most recent common ancestor of *bla*_KPC-2_-containing subclade B, while subclade C, defined by *bla*_KPC-4_ carriage, had an ancestral node dated at 1997 (PP = 1). bla_CTX-M-15_-containing subclade D had a most recent common ancestor which Bayesian reconstruction suggested dates to 2000 (PP = 1) and likely diverged from subclades B and C in 1984 (PP = 0.999).

### Repertoire of mobile genetic elements (MGEs) of ST171 and ST78 genomes.

MGEs unique to ST171 and ST78 genomes encoded a variety of adaptive mechanisms potentially enabling their success within hospital environments. Of eight predicted MGEs in ST171 reference genome 34978, three were distributed throughout both clades (Fig. 2A). A 30-kb genomic island (GI) (GI3; Table S3) unique to ST171 comprised 31 coding sequences (CDS), including a putative type IV toxin-antitoxin (TA) gene pair encoding cytoskeleton-binding toxin (CbtA) and cytoskeleton bundling-enhancing factor A (CbeA), which are thought to participate in cellular stress responses (18). Five additional genomic islands present only in ST171 clade B encoded several putative virulence factors, including arsenic resistance genes from the *ars* arsenic resistance operon (GI1) (19) and a cluster of genes encoding copper-binding and resistance proteins and belonging to the *cop* operon (GI2) (20). The ST171 reference genome also harbored six different phage regions, five of which were found throughout both clades (Fig. 2A). The 70-kb HK639 bacteriophage (Phage3; Table S3) putatively encoded an alpha-hemolysin, although this phage was limited to a small subset of ST171 isolates from New York City within clade B and was absent from other CREC clonal backgrounds.

Five of 10 genomic islands identified in the NR0276 ST78 reference genome were present in all ST78 isolates. These genomic islands encoded a variety of potential virulence factors, including a gene cluster encoding S-fimbrial adhesin proteins (GI1) (21) and the flagellin protein FliC and associated flagellar export and regulatory proteins (GI5) (22). However, the other five NR0276 genomic islands were present only in *bla*_KPC-3_-harboring clade A. This included GI6, a large genomic island (80 kb) composed of 61 CDS encoding the type II TA system PasT/PasI, which has been shown to enable pathogen persistence in limited-nutrient settings and in the face of oxidative and nitrosative stress in Escherichia coli (23). Genes involved in silver and copper sequestration and resistance, including components of the *sil*, *cus*, and *cop* operons, were also identified in a genomic island (GI9) associated with this clade (20, 24). The ST78 reference genome contained two phage regions, HK639 (Phage1) and the widespread 40-kb mEp390 bacteriophage (Phage2), which was present in most ST78 genomes as well as ST171 genomes.

### Plasmid-encoded antimicrobial resistance genes.

The presumed mechanism of carbapenem resistance in almost all CREC isolates regardless of clonal background was carriage of *bla*_KPC_-harboring plasmids (87%), although 1 CREC isolate harbored *bla*_NDM-1_ and 12 isolates lacked detectable carbapenemase genes. In order to determine the genetic context of *bla*_KPC_ in ST171 and ST78 isolates, we generated *de novo* hybrid assemblies of plasmids from representative isolates belonging to the major CREC ST171 and ST78 clades (Table S5). However, in non-ST171/ST78 isolates, particularly those harboring *bla*_KPC-2_ (*n* = 9), *bla*_KPC_ was associated with variable plasmid profiles and in most cases could not be assigned to plasmid backbones using short-read data.

**FIG 4:**
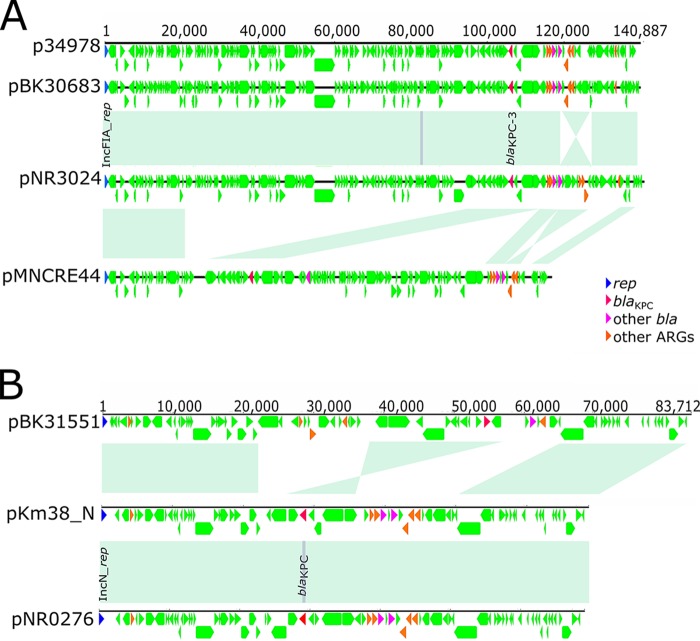
Genetic features of ST171 IncFIA plasmids. (A) The sequence of a representative 139-kb IncFIA plasmid harboring *bla*_KPC-3_ found in an ST171 clade B isolate, pNR3024, was compared to sequences of plasmids from E. cloacae complex reference isolates P34978 and pMNCRE41-2 and K. pneumoniae pBK30683. The areas of light green shading depict regions of homology, and resistance and other predicted genes are denoted as indicated. (B) A 69-kb IncN plasmid harboring *bla*_KPC-3_, represented by pNR0276, demonstrated close identity to the IncN pKm38_N plasmid, with the notable exception of a single SNP in *bla*_KPC_ consistent with a change in subtype. These IncN plasmids demonstrated regions of homology shared with *bla*_KPC_-_4_-harboring IncN plasmid pBK31551.

In ST171 clade A isolates, *bla*_KPC-4_ was present on an IncHI2A/IncHI2 plasmid (Fig. 2A). *De novo* assembly identified a large (315-kb) representative plasmid, pNR3082, which harbored *bla*_KPC-4_ within Tn*4401b*, an additional beta-lactamase gene, and aminoglycoside and sulfonamide resistance genes. The majority of ST171 clade B isolates putatively harbored an IncFIA plasmid harboring *bla*_KPC-3_, consistent with clonal spread (Fig. 2A). Representative 141-kb IncFIA plasmid pNR3024 contained the *bla*_KPC-3_ gene within Tn*4401d* as well as two additional beta-lactamase genes and several aminoglycoside and sulfonamide resistance genes (Fig. 4A). The backbone structures of pNR3024 were highly similar to those of p34798, an IncFIA plasmid derived from the 34978 reference genome, and pBK30683 in K. pneumoniae, which was previously shown to be widely distributed in hospitals in New York and New Jersey (Fig. 4; see also Table S5) (25). pNR3024 differed from these plasmids by 80 SNPs distributed in methyltransferase genes and genes associated with hypothetical proteins, a 98-bp insertion in the DNA polymerase III theta subunit of pNR3024, and an inverted 7.5-kb region distal to *bla*_TEM-1_ harboring an aminoglycoside resistance gene (Fig. 4). IncFIA plasmid pMNCRE44, which was identified among ST171 isolates in the northern Midwest (6), also shared a similar plasmid backbone consisting of three collinear blocks with close identity that resulted in approximately 60% homology with pNR3024 (Fig. 4), suggesting recent diversification.

Although ST78 included fewer CREC isolates, we found evidence for multiple distinct *bla*_KPC_-encoding plasmids in this clonal lineage (Fig. 3A). Most clade A CREC isolates harbored an IncN plasmid represented by pNR0276, a 69-kb plasmid containing *bla*_KPC-3_ within a Tn*4401b* transposon (Fig. 4B; see also Table S5). pNR0276 was also detected in three isolates belonging to unique STs (ST93, ST133, and ST269) and *hsp60* clusters, indicating a broad host range. pNR0276 closely resembled the IncN pKm38_N plasmid from a Klebsiella michiganensis strain harboring *bla*_KPC-2_ collected in 1997 (Fig. 4B) (26), differing by only 22 SNPs and a 210-bp transposase deletion. In subclades B and C, respectively, *bla*_KPC-2_ and *bla*_KPC-4_ were putatively located on distinct and yet related plasmids which shared an IncN replicon type and contained conserved backbone structures (Fig. 3A) (5, 8). The evidence for conserved elements in IncN plasmid harboring different *bla*_KPC_ subtypes was further demonstrated by alignment of pNR0276 to pBK31551 and *bla*_KPC-4_-harboring IncN plasmid derived from a K. pneumoniae isolate from a New Jersey hospital (27), which revealed the presence of three large collinear blocks with a total length of approximately 50 kb demonstrating >95% identity (Fig. 4B). We also found evidence in our collection for horizontal transfer of a pNR3024 plasmid from ST171 to ST78, as two closely related ST78 isolates obtained from the same patient 1 month apart (patient A, Table S1) differed only by the presence of this IncFIA plasmid.

10.1128/mBio.00542-18.4 TABLE S1  Clinical data and isolate susceptibilities of whole-genome-sequenced *Enterobacter* isolates. Download TABLE S1, XLSX file, 0.03 MB. Copyright © 2018 Gomez-Simmonds et al. 2018 Gomez-Simmonds et al. This content is distributed under the terms of the Creative Commons Attribution 4.0 International license.

To determine the *in vitro* transmissibility of plasmids from distinct CREC backgrounds, we determined transformation efficiencies for IncFIA and IncN plasmids. IncFIA plasmids were derived from a representative isolate within the major northern Manhattan cluster of ST171 clade A (pNR0011) as well as from outlying branches (pNR3040 and pNR3041). We also selected IncN plasmids from ST78 clade A (pNR3055) and from different STs (pNR1247 and ST454). We transformed these plasmids into E. coli DH10B cells and observed a significant difference in mean transformation efficiencies between the IncN pNR1247 plasmid and the IncFIA pNR0011_1 plasmid (7.2 × 10^4^ CFU/µl [standard deviation {SD}, 6.5 × 10^3^] versus 1.2 × 10^3^ [SD, 1.4 × 10^3^]; *P* = 0.01) (Fig. S3). Intermediate values were observed for IncFIA plasmids pNR3040 and pNR3041 and IncN plasmid pNR3055.

10.1128/mBio.00542-18.3 FIG S3  Transformation of IncFIA and IncN plasmids into E. coli DH10B cells. Transformation of three different IncFIA plasmids (pNR0011, pNR3040, and pNR3041) derived from ST171 isolates and two IncN plasmids (pNR3055 and pNR1247) from CREC ST78 clade A and an unrelated ST (ST454) demonstrated a significant difference in mean transformation efficiencies between pNR0011, which was derived from the main cluster of ST171 isolates at our northern Manhattan cluster, and pNR1247, which was derived from a sporadic CREC isolate (7.2 × 10^4^ CFU/µl [SD, 6.5 × 10^3^] versus 1.2 × 10^3^ [SD, 1.4 × 10^3^]; *P* = 0.01). Other isolates demonstrated intermediate mean transformation efficiencies. All experiments were performed in triplicate. The mean transformation efficiency for pUC19 control plasmids was 8.4 × 10^7^ CFU/µl (SD, 3.9 × 10^7^). Download FIG S3, PDF file, 0.03 MB. Copyright © 2018 Gomez-Simmonds et al. 2018 Gomez-Simmonds et al. This content is distributed under the terms of the Creative Commons Attribution 4.0 International license.

### CREC is a low-virulence pathogen.

**FIG 5:**
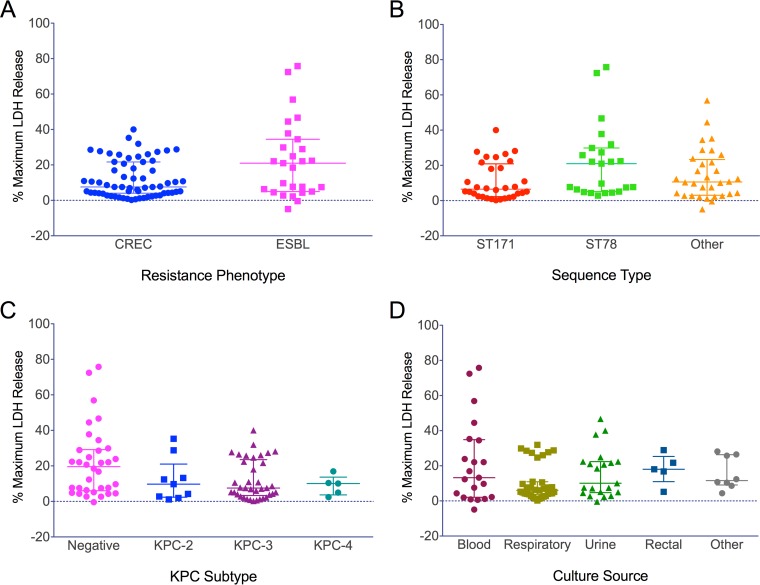
Differences in levels of cell killing among CREC and ESBL *Enterobacter* isolates. The data represent pooled median values for percentages of maximum LDH release compared to Triton X results after exposing murine macrophages to bacterial supernatant. The group median and interquartile range (IQR) are shown. Asterisks (*) denote significance (*P* < 0.05). (A) Overall, CREC isolates demonstrated significantly lower cytotoxicity than ESBL isolates (7.6% [IQR, 4.0 to 21.7] versus 21.0% [IQR, 5.0 to 34.5], *P* = 0.04). (B) There was a nonsignificant trend toward decreased cytotoxicity between ST171 and ST78 isolates (6.5% [IQR, 2.3 to 20.9] versus 21.0% [IQR, 5.1 to 29.9], *P* = 0.052), while other STs demonstrated heterogeneity in cell killing (median, 10.6 [IQR, 3.0 to 23.3]). (C) There was no significant association between cytotoxicity and *bla*_KPC_ subtype among CREC isolates (overall *P* = 0.1). (D) Likewise, levels of cell killing did not differ significantly across culture sources (overall *P* = 0.2).

To evaluate differences in virulence between CREC clones and between CREC and ESBL isolates, we employed a cytotoxicity assay using immortalized, bone marrow-derived murine macrophages. Macrophages exposed to cell-free bacterial supernatant derived from CREC isolates compared to ESBL isolates demonstrated a significant difference in the levels of cell killing (median versus maximum lactate dehydrogenase [LDH] release, 7.6% versus 21.0%, *P* = 0.04) (Fig. 5A). Compared to ST78 isolates, ST171 isolates showed reduced cytotoxicity, although the difference was not significant (6.5% versus 21.0%, *P* =0.052) (Fig. 5B). However, the subset of CREC ST171 isolates showed significantly lower toxicity than the ESBL ST78 isolates (6.0% versus 21.5%, *P* = 0.007). There were no significant differences in the levels of cell killing in comparisons of isolates from different ST171 and ST78 clades (overall *P* = 0.2). We also noted no significant differences in the levels of cytotoxicity between ST171 and non-ST171/ST78 isolates (6.5% versus 10.6%, *P* = 0.8), although the latter produced variable levels of cell killing (Fig. 5B). There were no significant differences in cytotoxicity among the isolates based on culture source or *bla*_KPC_ subtype (Fig. 5C and D).

## DISCUSSION

**FIG 6:**
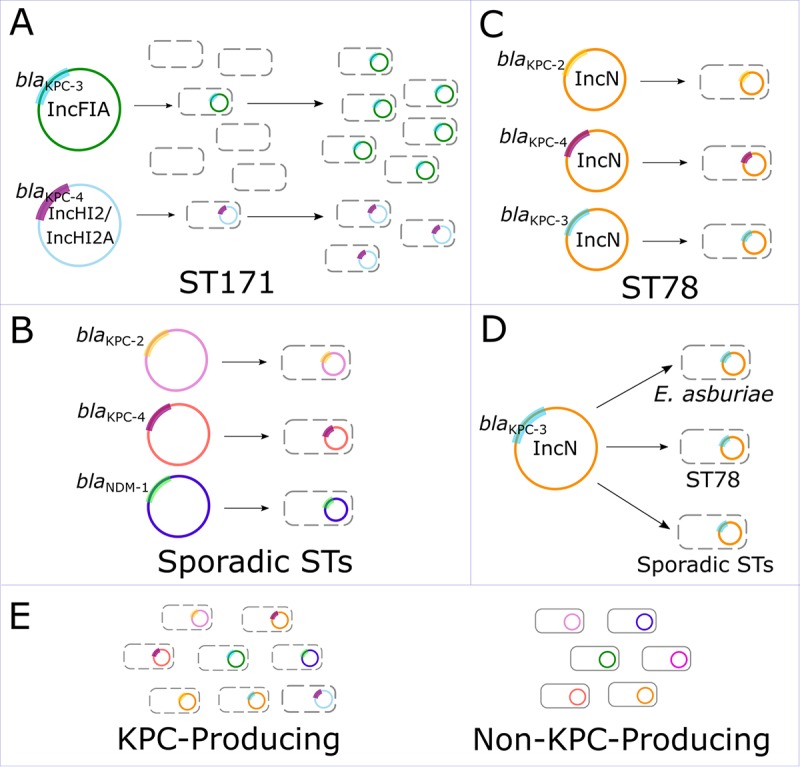
Proposed mechanisms of CREC evolution and dissemination. (A) Plasmid uptake followed by clonal dissemination. ST171 was highly associated with an IncFIA plasmid found to harbor *bla*_KPC-3_ in a representative isolate, suggesting that plasmid uptake followed by clonal dissemination was the dominant mechanism of carbapenem resistance in this predominant clonal lineage. (B) Uptake of *bla*_KPC_-harboring plasmids into a predominantly ESBL clone. ST78 demonstrated multiple independent plasmid uptake events. (C) Uptake of diverse plasmids into sporadic clones. Independent events of plasmid uptake into multiple different clones involved multiple different carbapenemase genes, including *bla*_KPC-2_. (D) Dissemination of a hypertransmissible plasmid. We detected IncN plasmids harboring *bla*_KPC-3_ in several different clonal sublineages, suggesting that this may be a highly transmissible plasmid. (E) Carbapenem resistance in non-carbapenemase-producing isolates. A subset of CREC isolates did not harbor *bla*_KPC_ or other known carbapenemases.

In this comprehensive collection of newly sequenced CREC isolates and published genomes, we found evidence for five putative mechanisms of CREC evolution and dissemination (summarized in Fig. 6). These included (i) independent acquisition of *bla*_KPC_ in ST171 by at least two distinct clonal sublineages with subsequent clonal spread and regional outbreaks; (ii) multiple instances of *bla*_KPC_ uptake into a dominant ESBL clone, ST78; (iii) distribution of an IncN plasmid among several different STs, indicating a possible hypertransmissible plasmid; (iv) uptake of a variety of carbapenemase gene-harboring plasmids into diverse STs; and (v) sporadic emergence of non-carbapenemase-producing isolates. Overall, the introduction of *bla*_KPC_ into multiple E. cloacae complex clones, including several distinct ST171 and ST78 sublineages, suggests that a combination of species-specific and external factors, such as antibiotic pressure, has contributed to the widespread emergence of CREC.

Our study provided robust evidence for the spread of two distinct ST171 clades across the United States, including a successful *bla*_KPC-3_-harboring sublineage demonstrating multiple foci of rapid, regionalized clonal proliferation in the Northeastern and northern Midwestern United States. Within the two clades, we also identified links between isolates from different regional and international locations, highlighting the potential for transmission of high-risk clones to contribute to the global spread of CRE. Although results of *in vitro* experiments suggested that ST171 is a low-virulence organism relative to other E. cloacae clones, ST171 isolates demonstrated remarkable cross-class resistance to antibiotics used commonly to treat Gram-negative infections, with the exception of amikacin. Notably, the acquisition of carbapenem and fluoroquinolone resistance determinants prior to widespread use of carbapenems and fluoroquinolones suggests that ST171 was already present in hospital settings prior to the mid-1980s and may have proliferated in the setting of rising antibiotic pressure.

Conversely, the emergence of CREC ST78 was characterized by multiple instances of sporadic uptake of *bla*_KPC_-harboring plasmids without evidence of extensive clonal spread. We identified three different CREC subclades defined by different *bla*_KPC_ subtypes and a large sublineage of isolates from northern Manhattan demonstrating update of the plasmid-mediated *bla*_CTX-M-15_ ESBL gene. Moreover, ST78 isolates harbored unique genetic factors that may make this clone particularly adept at thriving in nosocomial environments, such as metal-binding and resistance proteins and genes putatively enabling niche-specific colonization. Given that previous population analyses of MDR E. cloacae demonstrated that ST78 is a widespread, globally dominant ESBL clone (28, 29), this suggests that ST78 is a highly successful hospital-associated clone with a unique ability to accept plasmids harboring resistance genes.

In addition to high-risk CREC clones, our findings suggest that increased attention to plasmid-mediated transmission of *bla*_KPC_ is also warranted. We found evidence for recent horizontal transfer of a hypertransmissible IncN plasmid harboring *bla*_KPC_, which was identified in multiple CREC clones and demonstrated relatively high *in vitro* transformation efficiency. Among previous studies, IncN family plasmids were frequently implicated in dissemination of *bla*_KPC_ among multiple bacterial strains (30, 31). Interestingly, pNR0276 was found to be closely related to *bla*_KPC_-harboring IncN plasmids collected in the 1990s (26), further suggesting that it is a well-established plasmid backbone with the potential to enable multispecies spread of *bla*_KPC_. However, we also detected a wide diversity of CREC isolates harboring heterogeneous plasmids, particularly among *bla*_KPC_-harboring isolates. This may be driven by a growing hospital reservoir of *bla*_KPC_-harboring plasmids as well as intrinsic factors that enable rapid uptake and acquisition of novel plasmids in E. cloacae complex.

Our study had several notable limitations. Our sample size was limited to available clinical CREC isolates at our hospital system and to published CREC genomes and may have reflected oversampling of clonal outbreaks. In order to perform Bayesian phylogenetic and phylogeographic analyses, several assumptions were required, and inherent limitations of these techniques may have introduced uncertainty into our findings. The accuracy of the evolutionary timeline for both ST171 and ST78 would be improved by the availability of more-recent clinical isolates or of additional earlier isolates collected prior to the early 2000s. Although we demonstrated high node support at all primary branch points in our analyses, the availability of additional isolates may lead to improved resolution of both recent branches with low support and long branches which could not be resolved due to a lack of earlier isolates. Moreover, our phylogeographic analysis was limited by the availability of isolates from only a few health care facilities and, more broadly, by the inclusion of isolates from only the Northeastern and northern Midwestern United States. Ultimately, phylogeographic analysis will produce a tree rooted in one of the sampled locations. Therefore, despite our high statistical support for the idea of an origin of ST171 in Boston, the future inclusion of isolates from additional locations may either support or refute this finding. Lastly, our cytotoxicity model was limited to demonstrating cytolytic activity of bacterial supernatant and may not fully reflect the *in vivo* virulence profile of CREC clones. *In vivo* models and functional studies are needed to further explore the role of specific genomic determinants in shaping the distribution and success of ST171 and ST78.

In summary, here we elucidated diverse evolutionary pathways of high-risk and sporadic E. cloacae clones in a large data set of genomes from across the United States and international sites. The inclusion of long-read sequencing and *de novo* hybrid assemblies, including new genomic and plasmid reference sequences, minimized reference bias and enabled detailed analysis of lineage-specific MGE and resistance gene repertoires. Our results indicate that, rather than heightened virulence, it was likely the proclivity of E. cloacae to acquire and disseminate multidrug resistance determinants, coupled with its ability to adapt to nosocomial environments, that led to the wide dissemination of CREC. The clonal expansion of ST171 across the United States and its subsequent local proliferation point to the need to pay increased attention to this high-risk clone. However, ongoing uptake of *bla*_KPC_-harboring plasmids has the potential to generate new CREC sublineages in ST78 and other E. cloacae complex subtypes. This indicates a need for heightened surveillance efforts attentive to the potential for both local and international spread of high-risk clones and the emergence of new CREC sublineages.

## MATERIALS AND METHODS

### Selection of bacterial isolates.

We sequenced 125 MDR E. cloacae complex isolates collected between November 2009 and July 2016 at a tertiary care hospital in northern Manhattan (see Table S1 in the supplemental material). This collection encompassed all available CREC isolates, including 46 isolates for which clinical and genotyping data were described previously (7), and a subset of ESBL isolates belonging to ST78 or ST171 for comparison. We obtained clinical and microbiological data from a review of electronic health records and ascertained colonization status versus infection status using CDC National Healthcare Safety Network surveillance definitions (32). Antimicrobial susceptibilities were determined using Vitek2 or Etest (BioMérieux) as part of routine clinical care and interpreted using Clinical and Laboratory Standards Institute breakpoints (33). We identified an additional 104 publicly available CREC genomes for inclusion in the analysis (Table S2).

10.1128/mBio.00542-18.5 TABLE S2  List of publically available genomes included in this study. Download TABLE S2, XLSX file, 0.01 MB. Copyright © 2018 Gomez-Simmonds et al. 2018 Gomez-Simmonds et al. This content is distributed under the terms of the Creative Commons Attribution 4.0 International license.

10.1128/mBio.00542-18.6 TABLE S3  Contents of Enterobacter cloacae complex ST171 clade B 70-kb recombination region detected by Gubbins. Download TABLE S3, XLSX file, 0.01 MB. Copyright © 2018 Gomez-Simmonds et al. 2018 Gomez-Simmonds et al. This content is distributed under the terms of the Creative Commons Attribution 4.0 International license.

10.1128/mBio.00542-18.7 TABLE S4  Detection of mobile genetic elements in Enterobacter cloacae complex ST171 and ST78 isolates. Download TABLE S4, XLSX file, 0.01 MB. Copyright © 2018 Gomez-Simmonds et al. 2018 Gomez-Simmonds et al. This content is distributed under the terms of the Creative Commons Attribution 4.0 International license.

10.1128/mBio.00542-18.8 TABLE S5  Distribution of plasmids harboring blaKPC in northern Manhattan CREC isolates. Download TABLE S5, XLSX file, 0.01 MB. Copyright © 2018 Gomez-Simmonds et al. 2018 Gomez-Simmonds et al. This content is distributed under the terms of the Creative Commons Attribution 4.0 International license.

### Whole-genome sequencing and hybrid assembly.

Genomic DNA was extracted using a DNeasy UltraClean microbial DNA isolation kit (Qiagen). Index-tagged whole-genome libraries were sequenced using a HiSeq 2500 system (Illumina) and a MiSeq system (Illumina). We also conducted long-read sequencing for representative ST171 and ST78 isolates. DNA library preparation was performed using a Rapid Barcoding sequencing kit (Oxford Nanopore Technologies). Sequencing was then performed using a MinION DNA sequencer (Oxford Nanopore Technologies), and preprocessing of long reads with Poretools was performed as previously described (34, 35). Hybrid *de novo* assembly performed with SPAdes v3.10.1 (36) was followed by contig reordering using progressiveMauve in Geneious v10.0.5 (37) and annotation using Prokka v1.12 (38) and BLAST as previously described (35). Mobile genetic elements (MGEs) and prophage regions were identified by IslandViewer 3 (39) and PHAST (40), respectively. Gubbins v2.3.1 was used to identify possible recombination sites (41).

Isolates were classified by cluster membership (in clusters I to XI) based on *hsp60* housekeeping gene typing (14) as well as on multilocus sequence typing (MLST) (42), antimicrobial resistance gene profiling (43), and plasmid replicon typing (44) using SRST2 (45). To identify sublineage-specific MGEs, we used bowtie2 v2.3.1 (46) to align short reads against MGEs present in the ST171 and ST78 reference genomes, with no ambiguous matches permitted. Alignment lengths were tabulated using SAMtools v1.5 (47), and MGE presence was defined as alignment of ≥95% of MGE lengths. Putative plasmid presence was determined based on identification of replicon type and *bla*_KPC_ subtype using BLAST, as well as mapping of Illumina reads to reference plasmid sequences in Geneious v8.1.7 (48). Plasmids demonstrating at least 99% identity over 90% of the sequence length, including the replicon regions and the entire Tn*4401* transposon (49), were classified as closely related.

### Phylogenetic analysis.

We constructed phylogenetic trees by mapping individual genomes to ST171 and ST78 reference genomes to identify core genome single nucleotide polymorphisms (SNPs) using Snippy v3 (https://github.com/tseemann/snippy). Maximum likelihood phylogenetic analysis based on core genome concatenated SNPs was performed using RAxML v8.0.0 (50). Support for nodes was assessed using 100 rapid bootstrap inferences, and a final tree was selected through a maximum likelihood search under Gamma (50). Phylogenetic trees were visualized using iTOL v3 (51).

We performed Bayesian analysis to estimate the time to the most recent ancestor for ST78 and ST171. After all outliers were removed, a subset of ST171 and ST78 isolates displaying evidence of clock-like evolution as determined through TempEst (52) were used to estimate the substitution rate, time of emergence, and divergence dates within each clonal lineage using BEAST2 v2.4.7 (53–55). Using the HKY substitution Gamma site model with a relaxed lognormal molecular clock and coalescent Bayesian skyline prior, BEAST2 was run for 100 million generations, with sampling every 1,000 states and with isolate collection dates defined as available. Tracer v1.6 was used to inspect chain convergence and ensure an effective sample size (ESS) of ≥100 for all key estimated parameters (56). BEAST2’s TreeAnnotator v2.4.7 was used to identify the maximum clade credibility tree after 10% burn-in.

We also used Bayesian analysis to estimate the origins and phylogeographic spread of ST171 within the United States, with tip dates defined as described above and location assigned to NYC, New Jersey, Boston, or Minnesota/North Dakota. The BEAUti MultiTypeTree template was used to prepare the BEAST2 input using "location" as a discrete trait (57). The HKY substitution model and relaxed lognormal molecular clock were used, with the clock rate set to the ST171 BEAST analysis estimate (2e−3). The MultiTypeTree structured coalescent prior was used with lognormal distributions of kappa, population size, and rate matrix priors. The phylogeographic BEAST analysis was run for 400 million generations, with sampling every 10,000 states, and was repeated three times to ensure convergence of chains and sufficient ESS as calculated. LogCombiner v2.4.7 and Tree Annotator v2.4.7 were used to obtain a clade tree with maximum credibility after combining trees from all three runs with 10% burn-in. FigTree v1.4.3 was used for visualization of all BEAST trees.

### Microbiological characterization.

The cytotoxicity of cell-free culture supernatant obtained from a randomly selected subset of previously sequenced E. cloacae isolates (*n* = 90) was assessed *in vitro* using an immortalized murine bone marrow-derived macrophage (iBMDM) cell line. Once iBMDM cells were grown to confluence in a 100-mm-diameter petri dish, they were subjected to trypsinization and seeded the day before intoxication at 50,000 cells/well. Cultures grown overnight in Trypticase soy broth at 37°C with shaking at 2,469 × *g* were diluted to 1:100 and incubated for an additional 8 h. Bacterial supernatant was then collected by centrifugation and incubated with murine macrophages for 2 h at 37°C in 96-well flat-bottom plates. Cell viability was assessed by measuring release of lactate dehydrogenase (LDH), corrected for maximum LDH release by addition of Triton X to a control well. Cell viability assays were performed on 10 different colonies on 4 different days and pooled for analysis. Median differences in percentages of the maximum LDH release by Triton X were compared between isolate groups using the Mann-Whitney test or the Kruskal-Wallis nonparametric test with Dunn’s correction for multiple comparisons as appropriate, and data were visualized using GraphPad Prism v7.02 (GraphPad Software, Inc.).

IncFIA and IncN plasmids, including plasmids derived from major CREC ST171 and ST78 sublineages, were isolated and purified using a plasmid minikit (Qiagen) and were electroporated into competent E. coli DH10B cells (Invitrogen) using a Gene Pulser Xcell system (Bio-Rad Laboratories). Transformants were selected using LB agar plates containing 100 µg/ml carbenicillin. Gel electrophoresis was used to confirm plasmid sizes, and the presence of *bla*_KPC_ was confirmed by PCR using established primers (58). Transformation experiments were performed in triplicate, and efficiencies were calculated as the number of transformants per microgram of competent cells.

### Accession number(s).

Sequence data were deposited in the NCBI sequence read archive (SRA) (https://www.ncbi.nlm.nih.gov/sra) under accession numbers SRP099597 and SRP126514 (BioProjects PRJNA374677 and PRJNA421733). Individual BioSample numbers for each isolate are provided in Table S1. The NR0276, NR3024, and NR3082 hybrid assembly whole-genome shotgun projects have been deposited at DDBJ/ENA/GenBank under accession numbers PNXT00000000, PNXS00000000, and PNXR00000000, respectively. The versions described in this paper are PNXT01000000, PNXS01000000, and PNXR01000000. Novel housekeeping gene allelic variants were reported to the E. cloacae MLST database curator (http://pubmlst.org/ecloacae/).
