# Supplementary figures and images for: Genomic and Geographic Context for the Evolution of High-Risk Carbapenem-Resistant Enterobacter cloacae Complex Clones ST171 and ST78
Source: mBio. 2018 May 29;9(3):e00542-18. doi: 10.1128/mBio.00542-18 (PMC5974468; doi:10.1128/mBio.00542-18)

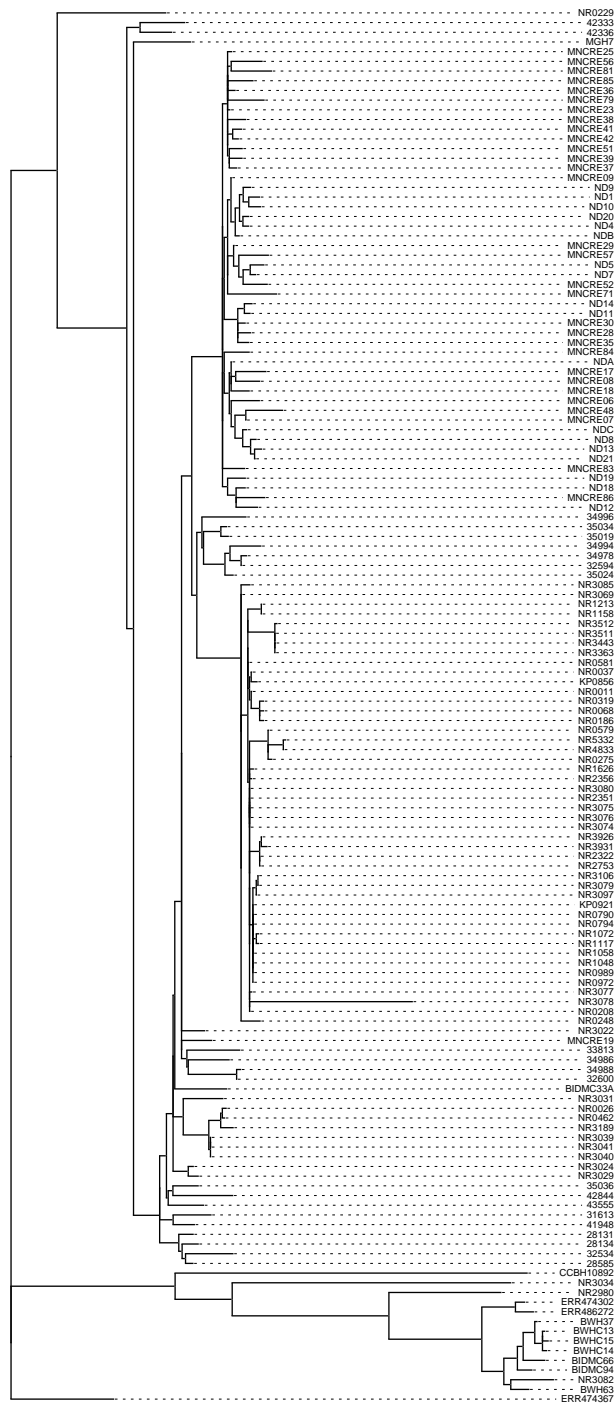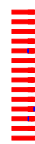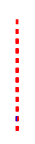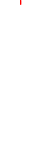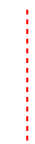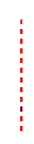

Supplement: FIG S1 [file mbo003183911sf1.pdf]

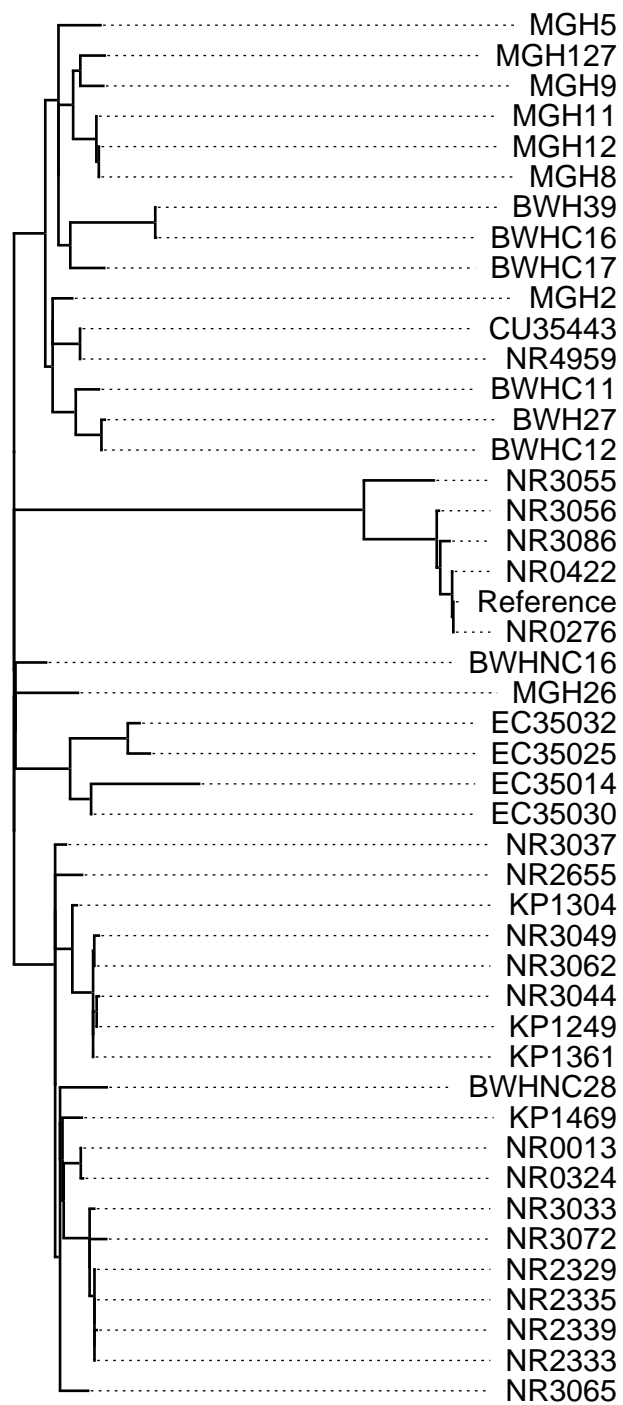

Supplement: FIG S2 [file mbo003183911sf2.pdf]

# Transformation efficiencies

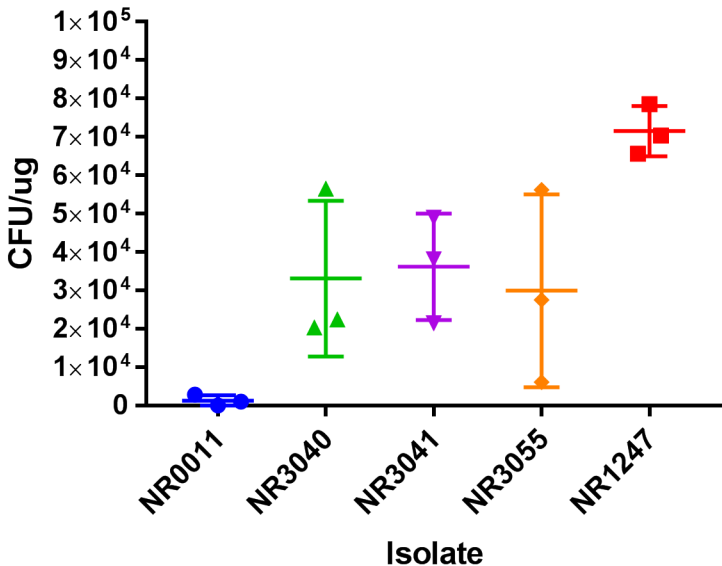

Supplement: FIG S3 [file mbo003183911sf3.pdf]
